# Cardiac Diseases and Disorders in Warfare

**Published:** 1915-12

**Authors:** Carey Coombs

**Affiliations:** Capt., R.A.M.C.(T.F.), 2nd Southern General Hospital, attached No. 32 General Hospital, British Expeditionary Force


### CARDIAC DISEASES AND DISORDERS IN WARFARE.

BY

CAREY COOMBS, M.D., M.R.C.P. Lond.,

*Capt., R.A.M.C.(T.F.), 2nd Southern General Hospital, attached No. 32 General Hospital, British Expeditionary Force.*

THE stress of work at the Southmead section of the 2nd Southern General Hospital has prevented any systematic investigation into the cardiac disturbances and lesions that have been encountered. Nevertheless, it may be worth while to report the most definite impressions that these matters have made on my mind.

First, there have been a fair number of cases of frank organic disease of the heart. I recollect but one example of the renal heart, in a man of about 40. His kidney lesion was obscure, and probably of long standing. His symptoms were cardiac, and it was on this score that he was invalided out. My own cases have only included one or two possible instances of cardiac syphilis, in the form of aortic valvular disease with some diffuse dilatation of the aorta. I have seen no case of aneurysm, though I believe one or two examples have been through the wards. Of post-rheumatic cases there have been quite a number. Many of these were obviously of long standing, but a few are traceable to rheumatic infection acquired, or at least manifested, since the outbreak of war. With one or two exceptions they have been cases of moderate severity, showing only a little enlargement of the heart and some mitral incompetence. The rheumatic group has also included several men with signs of aortic incompetence, and a few with evidence of mitral stenosis. I have seen no case of organic disease which can be directly traced to pure overstrain.

It might seem easy, at first sight, to decide what to do with these men. Anyone with organic heart disease should, it may seem, be discharged forthwith from the army. But since the prime consideration is whether or no the man is of most value to his country in the army or out of it, the problem is not quite so easy as it looks. Obviously men with pronounced cardiosclerosis associated with high tension are unfit for any kind of military service, and the same is true of cardiac syphilis. In both the symptoms are almost sure to get worse quickly, and the man will become useless to the army, whereas he is still fit for years of work at home. In cases of rheumatic origin, most men with pronounced valvular lesions are of more service to their country out of the army than in it. Occasionally one meets with a case of aortic regurgitation or of mitral stenosis in a man who may be usefully employed at home in training recruits, or even in general home service. In arriving at such a decision as this one is naturally influenced by the man's age, his term of service, and to some extent by his own inclinations in the matter. It is in patients with little or no dilatation of the heart, and a mere systolic bruit at the apex, that most of the difficulties arise. Many of these men improve considerably in hospital. Those who have no symptoms, whose lesion is found accidentally, may be safely returned to duty. For others, who complain of dyspnœa on exertion, and in whom there is manifest cardiac enlargement, home service may be at least tried. The chief point is that no man should be discharged merely because he has *signs* of cardiac disease ; *symptoms* are more significant than signs. Each case must be judged on its merits.

A much larger group of cases consists of those who fall for the most part under the heading of " D.A.H." We have seen a great many of these, far more than in civil practice. Unfortunately, I have been able to get at my notes on only twenty-five examples of this syndrome. With these as a basis I will briefly describe the condition which they exemplify.

*Ætiology.*—The average age was 24, 76 per cent. of the men being 25 or under. A similar percentage was derived from infantry units. More often than not the patients are of slender physique. Various causes were assigned by the men themselves. Several had felt symptoms during training. In one or two some special effort or exertion was blamed, while others thought it was the outcome of the general stress, physical and emotional, to which they had been exposed. Here it may be remarked that the cases are more frequently met with among the Gallipoli men than among those from Flanders. In two cases the onset was directly attributable to shell shock, and in two others it was as certainly consequent on a wound. One man thinks his symptoms are due to excessive smoking during convalescence. This tobacco factor is not by any means discoverable in all cases. Yet it seems to me that many of these men, or boys, as some of them are still, are smoking too much in hospital. Finally, the symptoms appeared in some cases during convalescence from some acute infection, such as dysentery, from typhoid fever, or malaria. Some of the men seem to me to owe a fraction of their subjective discomforts to the attention which they bestow on their hearts after they have been told —unwisely, as I think—that something is wrong with the heart.

*Pathogenesis.*—In the absence of any direct anatomical evidence, such data as are at present available must be pieced together. To begin with, there is no evidence in any of the cases I have seen of structural alteration in the heart itself, except of a mild, general dilatation, which—in some, at any rate—appears to be transient. On the other hand, there are distinct indications of a nervous or supracardiac origin. (1) The origin of the symptoms is often traceable to nervous shock or stress. (2) The pulse acceleration, which is so characteristic a feature of the syndrome, is subject in a majority to nervous excitation. (3) Certain of the more prominent physical signs, as will be shown below, are those associated in civil practice with states of nervous excitement. (4) The condition we are describing is often associated with other manifestations of an exhaustion neurosis. Of course, it is possible that there may be an intermediate toxic or even bacterial factor, and that nervous stress merely liberates some toxin or permits of some infection by which the symptoms are immediately provoked. Even so, however, the nervous factor is by far the more important.

*Symptoms.*—The onset may be abrupt, and traceable to some definite event; or gradual, even coming on during rest in hospital or convalescent camp. There are three chief symptoms, dyspnœa with exertion or excitement, palpitation, pain or other forms of cardiac discomfort, also increased by nervous or physical stress, and giddiness. Headaches and sleeplessness are also complained of by some. In a few the hands are cold and blue, but this is by no means universal. In many cases there are no subjective symptoms. If a number of wounded, or convalescents from dysentery, be examined, the irritable pulse and other characteristic features of the syndrome are found in quite a large percentage.

The pulse variations constitute the most remarkable and constant feature of these cases. These variations may be noted under three headings : (1) The average rate is quicker than normal (over 80 per minute in over 80 per cent. of the cases). (2) The rate of the pulse varies widely from day to day, and even from minute to minute. The ward charts show this. In one of my cases the rates charted varied from 80 to 136, in another from 72 to 100, from 56 to 92 in a third, and so on. (3) The pulse is accelerated wlth notable readiness by comparatively trivial physical and psychical stimuli. The physical stimulus most easily applied is a change of posture. In one of my cases the following remarkable variations were recorded. The rate while the patient lay flat in bed was 132 per minute. When he sat up the rate increased at once to 156, and stayed at this rate for at least two minutes. When he stood up by the side of the bed it increased to 192 per minute, and a true alternation of beats became apparent, together with pallor and giddiness. For several minutes after he lay down again this high speed persisted. This is an extreme case, but the same increase of pulse rate with alteration in posture, persisting abnormally, is highly characteristic. Emotional or psychic stimuli are rather more difficult to improvise. The patients are, nevertheless, well aware of the fact that excitement causes a notable increase in the rate of the heart. One curious example of this is to be found in the fact that the pulse rate as measured by the medical officer during his round is invariably faster than the average rate in the chart ; the interpretation, to which the patients themselves confess, being that the medical officer, with powers of discharge to duty, to subsidiary hospital, and so on, is a more formidable person than the sister, who is responsible for the pulse rates on the chart. The pulse is often irregular also, the arrhythmia being of the "sinus" type, *i.e.* it is due to causes operating through the supracardiac nervous mechanism, and not inherent within the heart itself. In a few cases extra systoles occur.

The *physical signs* are of some interest. The apex beat is forcible and diffuse, and extends too far to the left. Sometimes also it spreads downwards into the sixth interspace. There is, in fact, an apparently "inorganic" dilatation of the ventricles. Beside this there are limits of several kinds. These may be divided into three types: (1) A systolic murmur, brushing in quality, heard over a limited area, with its centre at or near the apex beat. This varies little with posture and respiration, but disappears after days, weeks or months. (2) A cardio-respiratory bruit heard at the apex, along the left border of the heart, loudest during inspiration and with the patient in the erect posture, diminishing and even disappearing when the patient lies down and also during expiration. The resultant sound heard on auscultation is comparable to an interrupted jet of air or vapour during inspiration, dying off into silence during expiration. This is a very familiar accompaniment of nervous states in civil practice. I have described it fully elsewhere, and given reasons for assigning to it a pleuro-pericardial origin. The other types of limit, (1) and (3), own, I believe, a similar source. They tend to vary from time to time as type (2) does, and they have the same "near-to-ear" quality. In type (3) the bruit is also systolic, and as in (2) varies in intensity with posture and respiration; but it is heard chiefly along the left border of the sternum, and at the pulmonic cartilage. Types (2) and (3) may be heard at the same time, or they may alternate in the same case. In all three types the bruit is apt to occur later in systole than an ordinary mitral regurgitant murmur, and it may even have a "pre-diastolic"

position in the cardiac cycle, running into and overlapping the second sound. In some cases no murmurs are heard at all. The first sound at the apex is nearly always accentuated.

*Diagnosis.*—The one great pitfall for the unwary is the ease with which these conditions are misinterpreted as examples of organic diseases of the heart. Of the twenty-five cases spoken of here no less than seven came to us with a diagnosis of "V.D.H."—a fact of some importance in intensifying the patient's nervous instability. I have several times written down a case at the first rapid routine examination as organic, and likely to need a medical board, only to find, three or four days later, that the signs were already modified, and the condition manifestly "functional." To guard against mistakes of this kind the following points should be recollected. (1) No one should be condemned as a case of "V.D.H." on the strength of a murmur alone. To do so is an act of malpractice. (2) Every murmur should be studied in its relation to respiration, and in at least two postures, the erect and the recumbent. (3) Just as certain bruits are characteristically organic, others—such as those described above under (2) and (3) in "Diagnosis"—are characteristically "functional." (4) Often one examination is not enough. Diagnosis should be reserved till several have been made at intervals of three or four days or longer. (5) The presence of the "irritable pulse" is always suggestive of non-organic disturbance.

*Prognosis.*—Many of these men improve quickly in hospital. A few, however, persist in defiance of rest and all other attempts at treatment. Their ultimate fate is uncertain. Certain physicians who watched cases of the kind during the Balkan wars assert that if they are not cared for they drift into a state of permanent arterial thickening with cardiac hypertrophy. My own experience is not extensive enough in point of time to justify any statement, but it has at least shown me how obstinate and damaging to the soldier's career this condition of irritable heart may be.

*Treatment.*—So far I have failed to find any drug that is of use, with the possible exception of bromides. The one measure that has any effect is rest. This does not necessarily imply a long period in bed, though it is well to keep all such men in bed for a few days if possible, for the sake of observation. After this, if the average pulse-rate is not over 100 they may be allowed to get up gradually, so long as they do not undertake any of the heavier ward work. The great point is to allow them a very long convalescence, of some weeks or even months. It is the mental rest thus secured that helps them most. If at the end of their time they have improved sufficiently they should be discharged to home service. Patients of this kind are never fit to go straight back to the nervous and physical stress of active service at the front. In some cases it is to be feared that they will never be fit for this. After a reasonably long probation they should be discharged from the army if shortness of breath still follows on moderate exertion and excitement. They will be more useful to the country making shells than being morally and physically knocked down by them.

One word may be said as to tobacco. On active service and during convalescence these men smoke a great deal. So far as is possible this should be controlled, especially in the case of the younger soldiers.

REFERENCE.

*Quart. J. Med.*, January, 1912, vol. v., No. 18.

